# A Framework Combining Delta Event-Related Oscillations (EROs) and Synchronisation Effects (ERD/ERS) to Study Emotional Processing

**DOI:** 10.1155/2009/549419

**Published:** 2009-07-08

**Authors:** Manousos A. Klados, Christos Frantzidis, Ana B. Vivas, Christos Papadelis, Chrysa Lithari, Costas Pappas, Panagiotis D. Bamidis

**Affiliations:** ^1^Laboratory of Medical Informatics, School of Medicine, Aristotle University of Thessaloniki, P.O. Box 323, 54124 Thessaloniki, Greece; ^2^Department of Psychology, City College, Affiliated Institution of the University of Sheffield, 54624 Thessaloniki, Greece

## Abstract

Event-Related Potentials (ERPs) or Event-Related Oscillations (EROs) have been widely used to study emotional processing, mainly on the theta and gamma frequency bands. However, the role of the slow (delta) waves has been largely ignored. The aim of this study is to provide a framework that combines EROs with Event-Related Desynchronization (ERD)/Event-Related Synchronization (ERS), and peak amplitude analysis of delta activity, evoked by the passive viewing of emotionally evocative pictures. Results showed that this kind of approach is sensitive to the effects of gender, valence, and arousal, as well as, the study of interhemispherical disparity, as the two-brain hemispheres interplay roles in the detailed discrimination of gender. Valence effects are recovered in both the central electrodes as well as in the hemisphere interactions. These findings suggest that the temporal patterns of delta activity and the alterations of delta energy may contribute to the study of emotional processing. Finally the results depict the improved sensitivity of the proposed framework in comparison to the traditional ERP techniques, thereby delineating the need for further development of new methodologies to study slow brain frequencies.

## 1. Introduction

During the past few years many studies have attempted to investigate the role of neuronal mechanisms involved in emotional processing [1]. Most of those studies employed Event-Related Potentials (ERPs) and focused on the detection of time-locked changes in the activity of large pools of neurons [2]. This approach has been mostly preferred because induced cerebral activity demonstrates an almost fixed time-delay to the stimulus onset, and it superimposes to the normal ongoing brain signals, which are regarded as additive noise. In these studies, typically a sufficiently large number of epochs with the same stimulus type are averaged in order to enhance the signal-to-noise (SNR) ratio. Consequently, this type of analysis aims at enhancing certain ERP components. However, this model can only roughly approximate reality, since it cannot deal with robust dynamical changes that occur in the human brain [3]. 

It is known that EEG activity is generated by sets of circuits [4]. The neuronal cells involved in these circuits are synchronized upon the appearance of a novel stimulus. Therefore, their activity is coupled and overall EEG coherency is enhanced. Thus, the system's complexity is reduced since it becomes more ordered. This results in the generation of rhythmic oscillations in various frequency bands which are superimposed on each other to form the compound event-related potential [5]. So, the analysis of ERP waves and their oscillations may promote our biophysical understanding of the brain functional networks and the investigation of both the global and the local characteristics of human brain activity. For instance, it is known [6], that there are particular events, which are time-locked to the event, but not phase-locked, and have an inhibitory effect to the alpha rhythm [7]. Consequently, the underlying cognitive processes cannot be thoroughly revealed using linear methods. Due to this limitation, frequency analyses are more appropriate, as long as, we assume that certain events affect specific bands of the ongoing EEG activity. This would result on the synchronization or desynchronization of underlying neurons exactly after the event (Event-Related Synchronization (ERS) or Desynchronization (ERD)) [7, 8]. The aforementioned reactions are time-locked to the event and concern specific brain waves. This type of spectral analysis is employed since selectively distributed oscillatory networks in the various frequency ranges control in an integrative way brain functions at every sensory and cognitive level. Cognitive processing of emotional visual stimuli involves several complex functions such as sensory processing, attention, decision making, and memory. Despite the potential interest of using such methodologies to emotionally evocative stimuli, research with these types of analyses employing the International Affective Picture System (IAPS) [9] is scarce. 

A few such studies have indeed shown that brain rhythms are associated with several cognitive processes. For instance, an increase in theta activity has been associated with initial learning improvement, which is typically followed by a decrease that reflects habituation processes [10]. A more relevant study, it has been shown that the amygdala produces theta activity in response to emotionally evocative stimuli [11]; whereas detection of unpleasant stimuli has been associated with theta synchronization in the hippocampus, which appears to be lateralized to the right hemisphere [12]. Moreover, slow waves, contribute to the detection of salient-infrequent-stimuli, and consequently, they contribute to the P300 response [13, 14]. Taken all together, these findings suggest that slow cerebral oscillations are suitable to study the cognitive processes related to emotions. 

Moreover, in the last few years, there is growing interest in investigating the neuronal mechanisms involved in the processing of emotionally evocative pictures [15, 16] using IAPS. IAPS pictures are rated in terms of valence and arousal. According to [17] emotional processing is mediated by two brain systems; the appetitive and the defensive. These two systems have evolved in order to assure the physical survival. The property “valence” refers to the direction of behavioral activation according to the motivational system induced by the stimulus. The property “arousal” represents the activation level elicited by the emotionally evocative stimulus [18]. The defense system is mainly active in unpleasant situations ranging from threat to melancholy and it is associated with “fight-or-flight” responses. On the other hand, the presence of pleasant situations like sustenance, procreation and nurturance activates the appetitive system. Therefore, the valence dimension refers to the system that is activated, while the arousal dimension refers to the intensity of this activation [19].

A few studies have investigated oscillatory modulation with visual emotional stimuli from the IAPS. In one of these studies [20], they investigated spectral responses in the gamma range by means of wavelet transforms, and found early effects (80 ms after stimulus onset) of the activity around 30 Hz which discriminated unpleasant stimuli from pleasant ones. Moreover, there was a later increase (480–550 ms poststimulus) in higher frequencies which appeared to be a reliable indicator of arousal. Theta activity has also been related to emotional stimuli, as early synchronization of theta activity has been reported, together with an interaction between valence and hemisphere for the anterior temporal regions [21]. In another study, it was found that theta ERS covaried with the stimulus intensity. Furthermore, the increase in theta activity was most pronounced over left anterior regions, and bilaterally over posterior regions [22]. Finally, the same study also reported that desynchronization of the medium alpha range was associated with attentional resources. The synchronization of the upper alpha in anterior cortical areas was also attributed to a greater cognitive involvement during processing of emotionally arousing stimuli [22]. To sum up, gamma, theta and alpha activities seem to be related with different aspects of the processing of emotional stimuli. 

However, to our knowledge, there is limited literature about delta wave activity and whether it is modulated during the emotional processing of complex visual scenes, like IAPS pictures, or not. This is in fact surprising, since there is some evidence which suggests that this wave may also play a role in processing of emotional stimuli. For instance, a research has reported a relationship between the P300 component and the delta frequency component [23]. Also, it is generally agreed, that the P300 is associated with unexpected or motivationally relevant stimuli [10]. For instance, emotional stimuli elicit a more pronounced P300 response than neutral ones [16]. Furthermore, studies have shown an increase of the activity in the delta frequency band during sexual arousal [24]. Another study [25] has indicated higher coherence of slow waves in central and posterior regions of the right hemisphere during sexual arousal induced by imagery. A summary of the studies that have been conducted with delta wave activity to investigate cognitive and emotional processes in healthy adults, and brain abnormalities in neurological disorders is shown in Table 1. It is important to notice that most of these studies used the linked earlobes montage, and the number of electrodes varied from 7 to 62.

The aim of the present study is to investigate the feasibility of using event-related delta oscillations to study brain processing triggered by visually complex emotional stimuli. Based on previous research, we hypothesize that delta wave activity, particularly in posterior brain areas, is mainly associated with arousal, whereas valence effects on delta activity will be mostly observed in anterior brain areas. Furthermore, and since our ERP studies have reported gender differences with the same stimuli [17], we also aim to check the replication of such findings in delta wave activity. That is, we suspect increased delta activity for females as compared to males, particularly for unpleasant stimuli. Furthermore, this piece of work aims at the investigation of laterality differences by extending the delta oscillations framework with the inclusion of delta synchronization analysis in terms of ERS/ERD. In addition, as the studying of delta wave activity was found to be more sensitive to arousal modulations of emotional stimuli than classic ERP peak studies [32], and it is linked with decision-making and salience feature detection properties [27], it is important to examine whether we obtain stronger valence effects of delta wave activity than with the classic ERP approach. In other words, we aim to compare herein effect sizes for both ERP [17] and delta wave analyses (the present study). Finally, a side methodological aim is to compare the different montage methods for spatial filtering. Although, most of the studies summarized above (Table 1) have employed the linked earlobes montage, only a few studies have actually compared the effectiveness of this method in relation to other methods such as the Common Average Reference (CAR) and (large) Laplacian (LAP). 

So, in the remaining of this paper, necessary background knowledge regarding the EROS extraction methodology, the artifact rejection approach and the ERD/ERS computation scheme are provided in Section 2. The results derived from the statistical analysis, which was performed on the average signals of each subject and for each emotional category are presented in Section 3. Finally, the contribution of the proposed framework to the estimation of the slow wave activity during emotional processing is discussed in Section 4. 

## 2. Materials and Methods

### 2.1. Subjects

Twenty eight healthy adults (14 males and 14 females) participated in the experiment (mean age of males 28.2 ± 7.5, mean age of females 27.1 ± 5.2). All subjects had normal or corrected to normal vision. Each participant signed an informed consent form prior to his/her participation and completed a short questionnaire. All participants were paid for their participation. An approval from the Ethical Committee of the Medical School of the Aristotle University of Thessaloniki, Greece, was granted for this study.

### 2.2. Stimuli

The participants passively viewed 160 emotional pictures, selected from the IAPS. The pictures were grouped in 4 conditions based on their valence and arousal ratings. The conditions were pleasant pictures with high arousal ratings (HVHA), pleasant pictures with low arousal ratings (HVLA), unpleasant pictures (Low Valence) with high arousal ratings (LVHA), and unpleasant pictures with low arousal ratings (LVLA). More details about valence and arousal ratings for each condition specific to gender are shown in Table 2. The experimental conditions were manipulated between blocks, thus there were four blocks of 40 photographs each.

### 2.3. Electrophysiological Recordings

During the presentation of each emotional block multichannel, Electroenchaphalogram (EEG) and Electrooculogram (EOG) were recorded with 500 Hz sampling frequency. Nineteen EEG electrodes were placed according to the 10–20 International System at sites Fp1, Fp2, F3, F4, F7, F8, Fz, C3, C4, Cz, T3, T4, T5, T6, P3, P4, Pz, O1, O2 with electrode resistance lower than 20 kΩ. Two EOG electrodes were placed above and below the left eye and another two were placed at the outer canthi of each eye. Two bipolar signals were calculated from these four electrodes, namely vertical EOG (VEOG) and horizontal EOG (HEOG) and used for effective EOG artifact rejection.

**Figure 1:**
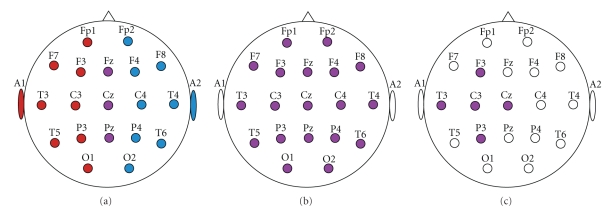
Illustration of the three different montage electrodes referencing methods. (a) Linked Mastoids Montage; left sites are referenced to the A1 while right site are referenced to A2, and the central sites are referenced to the half of the sum of A1 and A2. (b) C.A.R.; each site is referenced to the average of all electrode sites. (c) Large Laplacian; each site is referenced to the weighted average of their one-step neighbors.

Three different montages (Linked Mastoids, Common Average Reference and large Laplacian montage) (Figure 1) have been compared for optimal representation of cerebral responses. According to the linked mastoids montage, electrodes with odd indices were referenced to left mastoid and electrodes with even indices were referenced to right mastoid. Central electrodes (Fz, Cz, Pz) were referenced to the half of the sum of left and right mastoids. The Common Average Reference (CAR) which was computed according to the next formula:

(1) $$V_{i}^{\text{CAR}}=V_{i}-\frac{\sum_{j=1}^{n}V_{j}}{n},$$

where *j* is the number of electrodes and *V*_*i*_ is the potential of *i*th electrode. As for the third montage, the large Laplacian reference (LAP) has been employed, since the number of electrodes is restrictive for the small Laplacian reference. The following formula has been used for the calculation of the LAP:

(2) $$V_{i}^{\text{LAP}}=V_{i}-\overset{n}{\underset{j\in S_{i}}{\sum }}g_{ij}V_{j},$$

where

(3) $$g_{ij}=\frac{1/d_{ij}}{\sum_{j\in S_{i}}\left(1/d_{ij}\right)},$$

*S*_*i*_ is the set of electrodes surrounding the *i*th electrode, and *d*_*ij*_ is the distance between electrodes *i* and *j* (where *j* is a member of *S*_*i*_). The aforementioned distance is 3 cm for the small Laplacian montage, in contrast to large one, where the distance of two neighbor electrodes is 6 cm [33]. Linked mastoids were more effective in normalizing the data (see Section 3.1) as compared to the other methods, so it was further used for the purposes of this study. More detailed results are mentioned in Section 3.

### 2.4. Experimental Procedure

Participants were asked to sit on a comfortable armchair in front of a computer screen placed at 80 cm distance from their eye horizontal level. The experiment started with a 30-second recording, during which, participants were asked to keep their eyes open and look at the blank screen. This recording was followed by another one where participants were asked to keep their eyes closed. These recordings were taken to serve as baseline values. The experimental protocol was comprised of stimuli in the form of IAPS pictures, forming blocks of forty randomly selected pictures according to their arousal and valence ratings (Table 2). The order of the blocks was counterbalanced across participants. Each single epoch had 0.5 seconds prestimulus showing a white cross in the center of the screen and 2 seconds poststimulus period (1 second picture duration followed by 1 second of the white cross). At the end of the procedure the initial 30-second recordings, with eyes open and then closed, were repeated.

### 2.5. Event-Related Desynchronizations (ERD)/Event-Related Synchronization (ERS)

Sensory, motor, cognitive and emotional processing can affect the ongoing EEG by decreasing (ERD) or increasing (ERS) the synchrony of underlying neurons, so cerebral activity can be quantified using the ERD/ERS method which is described in [7]. ERD/ERS depict the percentage of band power changes during a test interval compared to a reference interval in a specific frequency band. There are a lot of different methods used today for quantification of ERD/ERS, which are summarized in [7]. The band power method [8] has been selected for the purpose of this study. According to this method each EEG signal for each channel was band-pass filtered in the delta frequency band (0.5–4 Hz), squared in order to calculate the delta band power, epoched and averaged over trials for each subject and for each emotion block. Finally in order to obtain the percentage of event-related changes in delta band power the following formula was used:

(4) $$\text{ERD}\left(\text{or}\;\;\text{ERS}\right)\%=\frac{R-A}{R}\cdot 100\%,$$

where *R* is the power of delta band in the reference interval (here 500 ms prestimulus) and *A* is the delta band power for the test intervals after the event (here 0–500 ms, 500–1000 ms, 1000–1500 ms, 1500–2000 ms after picture onset). For a more detailed description of this method see [10]. According to the aforementioned formula positive values (*R* > *A*) indicate that the test interval's band power is lower compared to the reference, which means that delta oscillations decrease their synchrony (desynchronize), and, therefore, ERD is obtained for positive values. Negative values (*A* > *R*) indicate neuronal synchrony in a similar way and they are obtained for ERS.

### 2.6. Artifact Rejection

**Figure 2:**
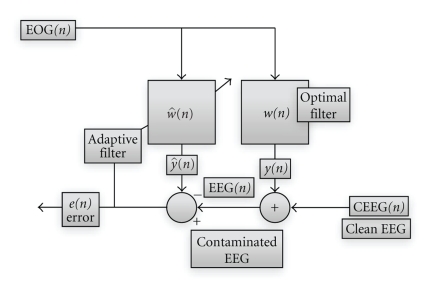
A block diagram of an adaptive filter. It is obvious that EEG(*n*) = *w*(*n*)EOG(*n*) + CEEG(*n*)*. * Adaption filtering is trying to adjust $\hat{w}(n)$ as close as possible to *w*(*n*), so our goal is to approach clean EEG as much as possible. $e\left(n\right)=\text{EEG}\left(n\right)-\hat{w}\left(n\right)\text{EOG}\left(n\right).\;$ It stands that *e*(*n*) → 0 when $\hat{w}(n)\to w(n)$ *.*

We have used the Least Mean Square (LMS) method, as it was proved to have better a performance among other widely used techniques for artifact rejection, based on Blind Source Separation (BSS) or regression methodology [34]. According to the LMS adaptive filtering procedure, the goal is to adjust the filter coefficients $\hat{w}(n)$ and make them approach the optimal filter coefficients *w*(*n*) as close as possible (Figure 2). The underlying idea of the LMS algorithm is to use a steepest descent algorithm to find the coefficients $\hat{w}(n)$ which minimize the objective function,

(5) $$F\left(n\right)=E\left\{{\left|e(n)\right|}^{2}\right\},$$

where *e*(*n*) is the error from the block diagram (Figure 2) and *E*{⋯} denotes the expected value. After the application of the steepest descent algorithm we have

(6) ∇F(n)=2E{∇e(n)e(n)},

where ∇denotes the gradient operator. For

(7) $$\;\begin{array}{c} \text{EOG}\left(n\right)={\left[\text{EOG}\left(n\right),\text{EOG}\left(n-1\right),\ldots ,\text{EOG}(n-p+1)\right]}^{T}, \end{array}$$

where *p* is the order of the adaptive filter, and ∇*e*(*n*) = −EOG(*n*) we have

(8) ∇F(n)=−2E{EOG(n)e(n)}.

∇*F*(*n*) is a vector orientated to the steepest ascent of the objective function, so we have to take the opposite direction of ∇*F*(*n*) for the minimization of ∇*F*(*n*). Thus we have the following equation:

(9) $$\begin{array}{ll} \hat{w}\left(n+1\right) & =\hat{w}\left(n\right)-\mu \nabla F\left(n\right)\\ & =\hat{w}\left(n\right)+2\mu E\left\{\text{EOG}\left(n\right)e\left(n\right)\right\}, \end{array}$$

where *μ* is the step size. Note that in most systems the expectation function *E*{EOG(*n*)*e*(*n*)} has to be approximated, and this can be achieved with the following estimator:

(10) $$\hat{E}\left\{\text{EOG}\left(n\right)e\left(n\right)\right\}=\frac{1}{N}\overset{N-1}{\underset{i=0}{\sum }}\text{EOG}\left(n-i\right)e\left(n-i\right),$$

where *N* is the number of samples used for the estimation. For *N* = 1 we have

(11) $$\hat{E}\left\{\text{EOG}\left(n\right)e\left(n\right)\right\}=\text{EOG}\left(n\right)e\left(n\right),$$

so the update algorithm is

(12) $$\hat{w}\left(n+1\right)=\hat{w}\left(n\right)+2\mu \;\text{EOG}\left(n\right)e\left(n\right).$$

### 2.7. EEG Processing

A band pass filter (0.5–40 Hz) and a notch filter at 50 Hz were applied to raw EEG signals. EOG signals were also notch filtered at 50 Hz for main line noise extraction but were band pass filtered in a different frequency band at 0.5–13 Hz. The artifacts originated from ocular activity were rejected offline with the use of the LMS adaptive filter algorithm. To obtain delta oscillations the EEG data were band-pass filtered in delta band (0.5–4 Hz) using Kaiser filter. After that, each EEG signal was epoched into 40 trials with duration of 2.5 seconds each (fixed length of 500 ms prestimulus and 2 seconds after picture onset) and averaged over these epochs to perform Event-Related Oscillation (ERO) analysis. Finally for each average signal, the ERD/ERS was calculated.

### 2.8. ERD/ERS Data Reduction

ERD/ERS values were averaged into 2-electrode clusters according to their hemisphere (Left Hemisphere: Fp1, F3, F7, C3, T3, P3, T5, O1; Right Hemisphere Fp2, F4, F8, C4, T4, P4, T6, O2) thereby facilitating the investigation of possible brain asymmetries.

### 2.9. Statistical Analysis

In order to investigate the evoked differences of delta oscillations to emotional stimuli, ERD/ERS mean values were submitted to a mixed 2 × 2 × 4 × 2 × 2 ANOVA with gender (male and female) as the between subject factor, and hemisphere (left and right), time intervals (0–500 ms, 500–1000 ms, 1000–1500 ms, and 1500–2000 ms), valence (pleasant and unpleasant) and arousal (high and low) as the within subject factors. Also 2 × 2 × 2 ANOVAs with gender as the between subject factor and valence and arousal as the within subject factors were conducted on the delta activity EROs of the six characteristic peaks (200–300, 300–400, 400–500, 600–800, 1100–1250, 1200–1500 ms after picture onset).

## 3. Results

As we mentioned above, the linked mastoids reference was more effective in normalizing the data as compared to the other two methods. In general, results showed that females had a stronger response to emotional stimuli as compared to males, in addition high arousal pictures provoke greater delta responses than the trials with low arousal pictures. Also delta responses provoked by unpleasant pictures were greater compared to the pleasant pictures. Finally, significant differences concerning the valence dimension were observed in the greater centro-frontal area, while the arousal was more discernible in the centro-parietal region.

### 3.1. Montage

**Figure 3:**
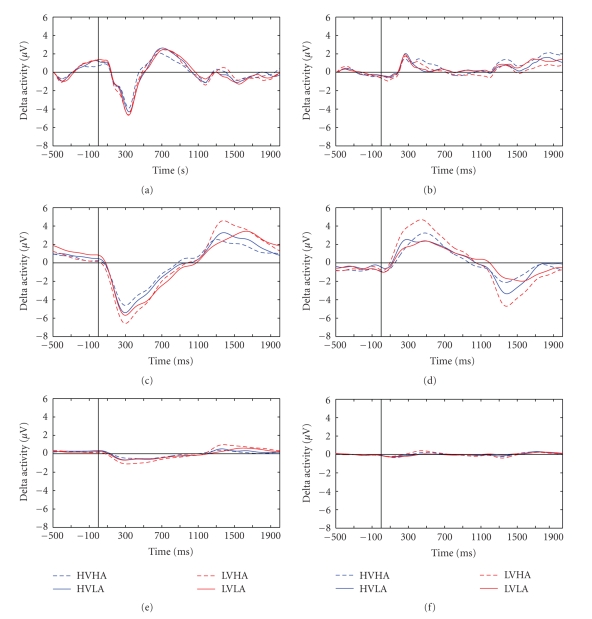
Comparison of different montage methods (Linked Mastoids-top-, Common Average Reference-middle- and Large Laplacian-bottom-) on the basis of delta activity waveform. Left: averaged delta oscillation waveform across all subjects and over all trials for Fz electrodes for all emotional stages separately. Right: delta oscillations in Pz, respectively.

As mentioned earlier, three different montages have been compared with regards to their effect in the ERD/ERS indices. Figure 3 illustrates delta waveform examples of the three montage methods. All reference techniques have similar overall features in terms of main peak responses. Otherwise the great scaling difference among the Large Laplacian montage and the rest methods is noticeable.

The results pointed out that the CAR and LAP approaches were not effective in normalizing the data; actually both montages increased greatly the mean square error (MSE) relative to the linked mastoids reference. For instance, the main effect of time interval was significant for all three methods, but the MSE was considerably increased for the CAR (*F*(3, 78) = 22.70, MSE = 2467660, *P* < .0001) and LAP (*F*(3, 78) = 43.78, MSE = 442632.50, *P* < .0001) methods in contrast to Linked Mastoids (*F*(3, 78) = 19.57, MSE = 1800.80, *P* < .0001). We also conducted the Kolmogorov-Smirnov test to check for normality, and found that data were not normalised with the CAR and LAP methods for many of the experimental conditions. This was not the case for the linked mastoid method, where all *P* > .05.

### 3.2. EROs

**Figure 4:**
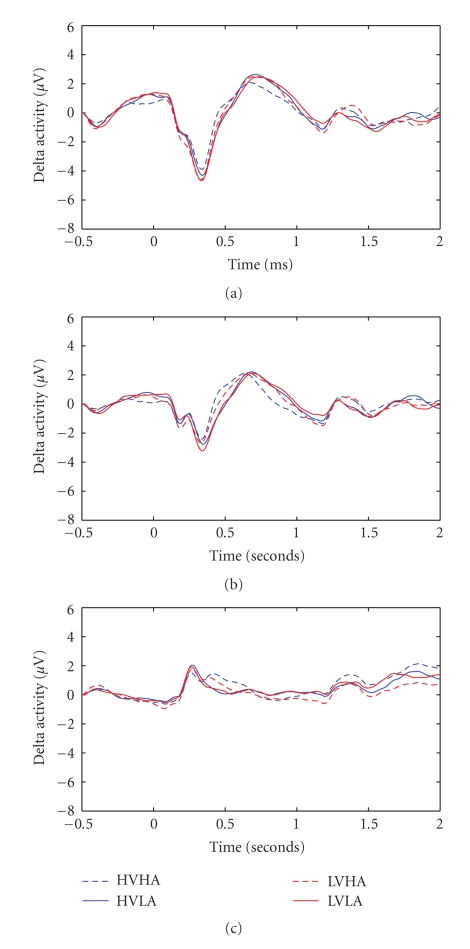
Illustration of delta oscillation over central electrodes (Fz-top-, Cz-middle- and Pz-bottom-). Averaged waveform across all the subjects for all emotional are used.

The averaged delta oscillatory activity from all the epoch segments was extracted for all the participants. The grand average waveform corresponding to the mean activity of all participants is visualized for each central electrode (Figure 4). The analysis presented herein is focused on the three central electrodes in order to study the differences among the four emotional categories and possible gender effects. Laterality issues are beyond the scope of the current EROs analysis.

The average delta activity contains six major peaks which are identified in the same temporal window for each block category. The selection of these windows was made in order to analyze early, late and very late cortical effects. The analysis is structured in a way to report only the significant results for each temporal window.

#### 3.2.1. Positive Peak 200–300 ms

There was a significant arousal by gender interaction (*F*(1, 26) = 5.824, *P* = .023) on the central electrode (Cz). Planned *t*-test revealed significant differences between the low arousal (1.0766 *μ*V) and the high arousal (−0.0938 *μ*V) conditions only for the female group (*t*(13) = −2.643, *P* = .020). No other effect reached statistical significance, *P* > .05.

#### 3.2.2. Negative Peak 300–400 ms

Results revealed significant main effects of valence (*F*(1, 26) = 4.378, *P* = .046) and gender (*F*(1, 26) = 7.136, *P* = .013) on the centro-frontal area. More specifically, unpleasant stimuli elicited greater responses than pleasant ones (−7.588 and −8.177 *μ*V, resp.), and females produced greater responses relative to males (−6.469 and −9.297 *μ*V, resp.). On the central electrode (Cz), there was a marginally significant main effect of gender (*F*(1, 26) = 4.01, *P* = .056), that is, females showed stronger responses (−6.495 *μ*V) than males (−4.212 *μ*V).

#### 3.2.3. Positive Peak 400–500 ms

Results on the central electrode exhibited a highly significant main effect of arousal (*F*(1, 26) = 17.209, *P* < .001); the delta activity was stronger for the high arousal condition (0.686 *μ*V) relative to the low arousal (−0.455 *μ*V) condition during this temporal window. Similarly, for the parietal electrode only the main effect of arousal reached statistical significance (*F*(1, 26) = 68.511, *P* < .0001), with high arousal condition eliciting much stronger responses than the low arousal condition (2.0043 and 0.8763 *μ*V, resp.). 

#### 3.2.4. Positive Peak 600–800 ms

Only the valence by gender interaction reached statistical significance (*F*(1, 26) = 5.398, *P* = .028) on the frontal electrode. Planned *t*-tests showed significant differences between males (1.59) and females (2.69) (*t*(26) = −3.069, *P* = .005) only for the unpleasant pictures, however there were no differences between male and female groups for the pleasant pictures, *P* > .05. In addition, there were significant differences between pleasant and unpleasant pictures (2.0742 and 1.5091 *μ*V, resp.) (*t*(13) = 2.641, *P* = .02) only for the male group. 

Table 3 summarizes the statistical analysis for both ERP [17] and delta wave methodologies, which were performed on the same data. As it can be seen the proposed framework appears to be more sensitive to arousal effects of the emotional stimuli. The proposed framework revealed Gender and Arousal by Gender effects in Fz with higher *F*-values, which means better sensitivity/specificity. Also the valence and the arousal effects in Cz and Pz respectively are superimposed by the use of the proposed framework analysis. At the current delta activity framework revealed more statistical significant effects in contrast to traditional ERP analysis (see Table 3). 

### 3.3. ERD/ERS

All ERD/ERS mean values for delta oscillations are negative, so it can safely be deduced that delta oscillations are synchronized after emotional stimuli. It has to be noted that lower ERS values (from an algebraic point of view) denote higher increases of delta band powers.

Results showed a significant main effect of time (*F*(3, 78) = 19.57, *P* < .0001). Scheffe posthoc comparisons showed significant differences between the first interval (−40.083) and the three remaining intervals (mean ERS value equal to −19.9565, −18.98 and −10.4989, resp., all *P*-values <.0001). No other comparisons reached statistical significance, *P* > .05. That is, delta oscillations increased their band power after the stimulus onset (0–500 ms), dropped significantly in the second interval (500–1000 ms) and remained almost stable in the next two intervals (1000–1500, 1500–2000). Also, the main effect of arousal was significant (*F*(1, 26) = 4.99, *P* = 0.034). High arousal stimuli (−25,474) produced greater synchronization of delta wave than low arousal pictures (−19,285). In addition, the following 2-way interactions were significant: gender by time interaction (*F*(3, 78) = 3.74, *P* = .014), and valence by arousal (*F*(1.26) = 4.55, *P* = .043). The gender by time interaction, evident only right after stimulus onset (0–500 ms interval), was due to a stronger response for female participants (−50%) than for male participants (−30%). Most importantly, these interactions were further modulated by a significant 5-way interaction, gender by hemisphere by time by valence by arousal interaction, (*F*(3, 78) = 3.01, *P* = .035).

In order to analyze further the 5-way interaction, we conducted two separate ANOVAs for each gender group with hemisphere, time, valence and arousal as the within subject factors (Figures 8–11).

### 3.4. Males

For the male group, results showed a significant main effect of time intervals (*F*(3, 39) = 4.83, *P* = .006). Scheffe posthoc comparisons exhibited significant differences only between the first (−30.3349) and the last interval (−12.3907), *P* = .008. Also, the main effect of arousal was highly significant (*F*(1, 13) = 5.63, *P* = .034). That is, trials with high arousal pictures (−25.1821) provoke greater synchronization of delta rhythm as compared to trials with low arousal (−15.8206) pictures.

Also, the following interactions were significant in the male group: hemisphere by valence, (*F*(1, 13) = 4.62, *P* = .05) (Figure 9), and hemisphere by time by valence by arousal, (*F*(3, 39) = 2.61, *P* = .065) (marginal). The hemisphere by valence interaction was due to significant differences between the left (−15.25) and right (−23.31) hemisphere for the unpleasant pictures, (*t*(13) = 2.11, *P* = .05); whereas no differences were found between the two hemisphere conditions for the pleasant pictures, *P* > .05. 

Finally, in order to analyze the 4-way interaction, we conducted four separate ANOVAs for each time interval with hemisphere, valence and arousal as the within subject factors.

#### 3.4.1. Interval 1 (0–500 ms)

The analyses for the time interval of 0–500 ms revealed a significant main effect of hemisphere, (*F*(1, 13) = 5.53, *P* = .035). Mean ERS value for left hemisphere was −26.6563, whereas right hemisphere's ERS was −34.0134. No other effect or interaction reached statistical significance, *P* > .05.

#### 3.4.2. Interval 2 (500–1000 ms)

In the second interval, 500–1000 ms, there was a significant main effect of arousal, (*F*(1, 13) = 4.98, *P* = .043). That is, there was a greater synchronization of delta activity for the high arousal pictures (−27.4742) than for the low arousal pictures (−16.1314). 

#### 3.4.3. Interval 3 (1000–1500 ms)

In the third interval, 1000–1500 ms, there was a significant main effect of arousal as well, (*F*(1, 13) = 9.07, *P* = .01). As it was observed before, there was a greater synchronization of delta activity for the high arousal pictures (−23.2588) than for low arousal pictures (−11.6904). In this third interval there was also a significant hemisphere by valence interaction (*F*(1, 13) = 8.31, *P* = .013) (Figure 10). That is, whereas synchronization of delta rhythm was greater for the pleasant picture (−18.34) than for the unpleasant pictures (−14.38) in the left hemisphere, the opposite pattern was observed for the right hemisphere (−14.57 versus −22.61).

#### 3.4.4. Interval 4 (1500–2000 ms)

Finally, in the analyses of the fourth interval, 1500–2000 ms, none of the main effects or their interaction reached statistical significance, *P* > .05. 

### 3.5. Females

The analyses for the female group revealed a significant main effect of time interval as well (*F*(3, 39) = 15.69, *P* < .0001). Scheffe posthoc comparisons showed significant differences in the mean ERS value between the first interval (−49.8315) and other three intervals (−18.1076, −20.4854, −8.6071, resp.; *P* < .0001). Furthermore, the interaction time by valence reached statistical significance (*F*(3, 39) = 4.27, *P* = .012). To analyze this interaction we conducted two separate ANOVAs for each valence condition with time as the within subject factors. Results showed a significant main effect of time for both pleasant and unpleasant conditions ((*F*(3, 81) = 14.683, *P* < .0001) and (*F*(3, 81) = 15.829, *P* < .0001), resp.). Scheffe posthoc comparisons revealed significant differences between the first and the other three intervals for both the pleasant, (−37.501, −19.094, −21.668, and −11.442; *P* < .0001), and the unpleasant condition, (−42.665, −19.094, −21.668 and −9.555, resp.; *P* < .0001). However the interaction was due to a greater difference between the first and the second interval for the unpleasant pictures relative to the pleasant ones.

## 4. Discussion

**Figure 5:**
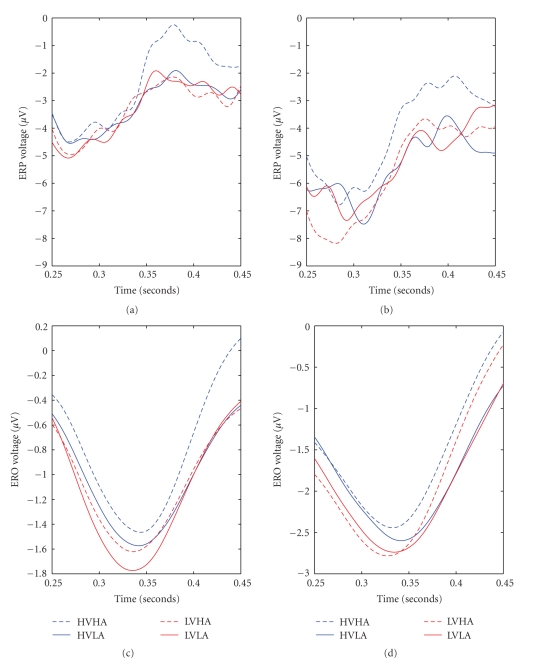
ERPs versus EROs findings for Fz electrode during the P300 response. Averaged waveforms across all subjects (males (left) females (right)) for all emotional stages. Top are depict ERPs while EROs are depicted in two bottom subfigures. All are in the Fz site between 250–450 ms.

**Figure 6:**
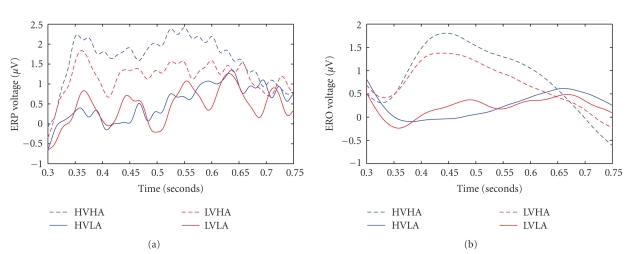
ERPs versus EROs findings for Pz electrode in the 300–750 ms time interval. Averaged waveforms across all the subjects for all emotional stages. ERPs are depicted in (a) while EROS are illustrated in (b).

**Figure 7:**
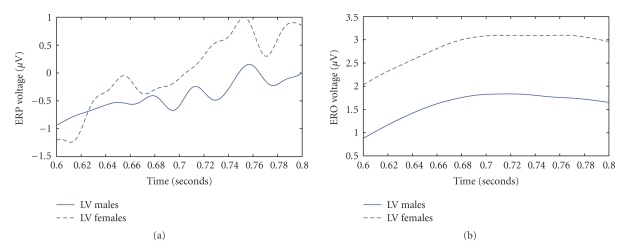
ERPs versus EROs findings for Fz electrode in the 600–800 ms time interval. Averaged waveforms over the gender (solid line: Males, dashed line: Females) for the low valence category. ERPs are depicted in (a) while EROS are illustrated in (b).

**Figure 8:**
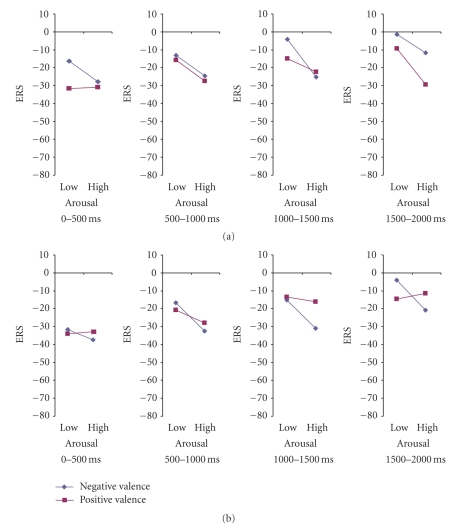
Five-way interactions of ERS index according to valence and arousal dimensions for males in (a) left and (b) right hemisphere.

**Figure 9:**
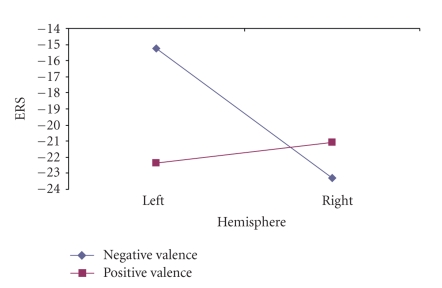
ERS dependence on the stimuli's valence. Note the laterality differences for males.

**Figure 10:**
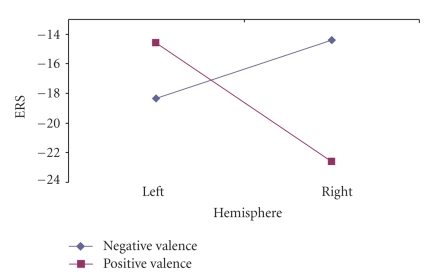
Valence by hemisphere interactions for males in third test interval.

**Figure 11:**
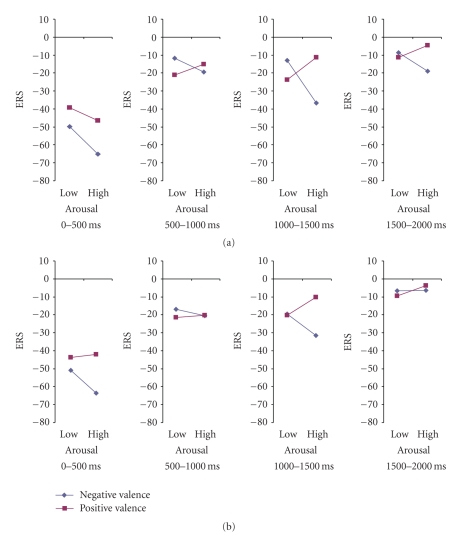
Modulation of ERS index according to valence and arousal dimensions for females in (a) left and (b) right hemisphere.

The major methodological aim of this paper was to investigate the feasibility of using a combination of event-related delta oscillations and delta synchronization analysis in terms of ERS/ERD in order to study emotional brain processing triggered by visually complex emotional stimuli. We have provided evidence herein that this kind of analysis is probably more sensitive to study not only arousal but also valence modulations of emotional stimuli than classic ERP peak studies. To provide such evidence, we compared effect sizes for both ERP and delta wave analyses. The resulted improved sensitivity of the proposed framework is given in both quantitative and qualitative terms, that is, on one hand, by the statistical analysis for both ERP and delta wave methodologies as depicted in Table 3; moreover, the differences between the two methodologies are qualitatively illustrated by Figures 5–7. According to Figure 6, the arousal modulation of the posterior areas is more intense when adopting delta oscillatory methodology in contrast to traditional ERP analysis. Similarly, the proposed framework is more sensitive in revealing the gender effect and valence modulation of the anterior areas as depicted in Figure 7. Finally, Figures 5  and 6  highlights the gender by valence and the gender by arousal interactions which also occur in the frontal lobe.

### 4.1. ERD/ERS

Delta oscillations are categorized among the brain's natural oscillations, which generally provide basic links to cognitive functions by integrative control at all levels [4]. According to [35], delta responses are evoked in the entire scalp of the human brain by sensory stimulation. The activity in this frequency band is related to signal matching, decision making and surprise [36]. Our results concerning this type of analysis indicate the important role of the delta activity as a marker of emotional processing. As already commented above, this type of analysis is more sensitive than the classic ERP peak approach; this is also supported by the fact that interactions like the valence by arousal interaction according to the subject's gender are revealed by the framework proposed here. Specifically, female participants exhibited stronger responses than males, particularly right after stimulus onset. Also, in general, high arousing and unpleasant pictures provoked stronger responses. In addition, the effects of valence and arousal in delta oscillations were modulated by gender. As expected, when an ERD/ERS analysis has been conducted, hemispheric differences have been found for the aforementioned effects in the male group. In a previous study [37] authors remark the contribution of delta waves in emotional modulation by visual inspection of emotional face expressions. The results presented in this paper demonstrate the participation of delta oscillations in emotional processing, in agreement with what has been shown in that study [37].

The main findings for the ERD/ERS analyses were the following: high arousal pictures provoked greater ERS responses of delta oscillations than low arousal pictures, which is in line with previous literature [10, 22], and this was mostly the case for the males participants. Moreover, females showed a greater ERS response as compared to males not only right after stimulus onset (0–500 ms interval), but also during the whole trial. Both males and females showed an effect of valence on ERD/ERS responses. Though, this effect interacted with hemisphere in males. On the other hand, in females, regardless of hemisphere, unpleasant pictures provoked a greater ERS values relative to pleasant pictures, whereas in males this was the case only for the right hemisphere. In the left hemisphere of the male group, ERS exhibited greater values for the pleasant pictures relative to the unpleasant. The prevalence of the right hemisphere in the emotional processing was also supported by a stronger initial (0–500 ms interval) synchronization in the right hemisphere relative to the left hemisphere.

Finally, results showed that the time course of these effects differed for males and females. Females showed a significant effect of the emotional valence of the stimulus only right after onset, whereas the effect of valence on ERS appeared much later (in the 1000–1500 ms interval) in males. The effect of arousal on ERS response was observed relatively soon (500–1000 ms after stimulus onset) in males. These findings suggest that there is a slower synchronization of delta oscillations in response to emotional stimuli for males as compared to females.

The initial stronger and faster response of females to emotional stimuli, as shown in the ERS analyses, replicate previous findings [38, 39], that have shown gender differences in cognitive tasks. Thus, it is generally agreed that male's performance is better on spatial tasks, whereas females perform better on emotion-related tasks [40–42]. These results suggest that this difference may not only be due to cultural influences but may also reflect gender differences concerning information processing in the brain. There were also gender differences in arousal. While males showed greater ERS values for HA pictures relative to LA pictures, there was not a significant effect of arousal for females.

As it is expected, ERS analyses were sensitive to brain asymmetry in emotional processing as well. Clearly, the results summarized above, support the dominance of the right hemisphere in emotional processing but only for males. In females, the emotional response was not lateralized. It is known that specific cognitive processes are lateralized either to the left or right hemisphere in males [43]. In contrast, cognitive processes are not so strongly lateralized in females, possibly because of the anatomical differences in the corpus callosum; it has been reported that women have a larger callosal size, which would enhance inter-hemispheric transfer, and would result in stronger bilateral processing as compared to males [43]. This may explain why we found brain asymmetry effects on ERS responses in males but not in females. Our finding regarding right hemisphere prevalence in emotional processing, particularly for unpleasant-threatening stimuli, is also in line with already published literature (see [44] for a review). On the other hand, our results show that the left hemisphere appears to be dominant for the processing of pleasant-positive-stimuli. As Davidson has shown [45–47], the LH is associated with more positive emotions in contrast to the RH, which is more involved in negative stimuli [48].

### 4.2. EROs

The most consistent finding was that female participants exhibited in general greater responses than male participants, and this finding was true for early and late components. The literature about gender differences in emotional processing is limited and focuses mainly on brain asymmetries employing event-related potentials. For instance, a recent study [49] investigated memory processing of faces that were classified as neutral, friendly and unfriendly. The ERP analysis demonstrated larger amplitudes in female participants relative to male participants. These differences were present during both early processing, as indicated by N300 and N400 components and late processing which lasted until the P600. Thus, the present study generalizes this finding to complex emotional stimuli, using a different measure of brain activity. In the present study, gender differences were stronger at the early negative peaks (N300, N400). Also, in agreement with this study [49], we observed later gender effects by positive deflections approximately 600–800 ms and 1200–1500 after stimulus onset. A more recent study [50] used simple light stimulation in order to investigate gender differences during the various frequency bands of the human EEG. The results showed that the delta response amplitudes for women were significantly higher than for men over occipital, parietal, central and temporal electrode locations. Consequently, the specific frequency band has a key role in the investigation of gender differences in the processing of emotionally stimuli. At last, gender differences in emotional processing can be explained in terms of differences in phyletic memory [51]. This memory has genetic origin and is based on the evolution theory.

The second main finding was that event-related delta oscillations were also modulated by the valence of the stimulus. In general, unpleasant pictures provoked greater responses than pleasant pictures did, although this effect was sometimes modulated by gender. Also, these effects were stronger and most consistent at the frontal electrodes (e.g., N300–400, P600–800, and N1100–1200 components). This finding agrees with previous work [52] that has shown that areas in the frontal cortex are activated by the valence dimension of the emotional stimuli. There is also converging evidence from neuropsychological studies, which supports a deficit in the processing of pleasant stimuli after damage to the left dorsolateral area [53], whereas bilateral lesions of the ventromedial prefrontal cortex are associated with inability to anticipate the rewarding consequences of an action [54]. Neuroimaging studies with healthy adults have also reported [55] activation in the right frontal region during withdrawal-related negative affective states. However, despite the great amount of studies investigating the laterality of emotional processing in the frontal cortex, the function of the medial frontal cortical structure has not been studied so thoroughly. The present study suggests that the analysis of temporal changes occurring in this area reveals an effect of the emotional valence of the stimulus during the early processing (N300–400 component), since the activation of the defense system (unpleasant pictures) elicits greater delta activity in comparison to the activation of the appetitive system (pleasant pictures). 

Finally, the results suggested that the positive peak that occurred almost 350 ms after stimulus onset mainly on parietal locations resulted in a strong arousal effect. Arousal effects on delta oscillations were also found for later positive (P400–500 and P1200–1500) and negative components (N1100–N1200). A substantial portion of P300 variation appears to be caused by fluctuations in the arousal state of the participants [56]. A more recent study using IAPS pictures, demonstrated that the emotional stimuli elicit a more positive wave in the P300 area than neutral stimuli. Further evidence is provided by studies that showed enhanced P300 responses to alcohol and smoking-related cues in alcoholics and smokers respectively [57, 58]. On the other hand, time-frequency analysis of task-related and rare stimuli yielded a later delta coefficient with a parietal predominance [59]. These findings are further supported by findings that suggest the contribution of delta activity to P3b expression [60]. Furthermore, a more technical study [61] demonstrated that P3b components have a more centro-parietal distribution. Indeed the results reported above mention a main effect of arousal 450 ms after stimulus onset. However, the latency of this response is delayed in comparison to the ERP occurrence and it also follows the parietal delta response.

The results of this piece of work provide some evidence towards the confirmation of the authors' hypothesis, that human emotional state and its associated brain processing related to and affects delta frequency activity. However, the relatively small number of electrodes used in the experimental recordings pertains to certain limitations [4, 37], since the spreading effect of the low conductivity skull cannot convey on detailed information of the scalp distributed potentials. So, there is a need for further research in this field with more accurate recordings, which will disclose effectively the scalp distributed phenomena occurred by emotional visual stimuli. It is also expected that the full intraband comparison will delineate the human brain emotional processing characteristics in a more comprehensive way. Nevertheless, the results presented here regarding the affection of delta oscillations by emotional stimuli cannot be overlooked.

In conclusion, to the best of our knowledge, this is the first study concerning the relation of delta oscillations (in terms of peak amplitude analysis and synchronization effects) and emotional processing triggered by visual stimuli from the IAPS collection. The results obtained here denote the important role of emotional processing in delta wave modulation. However, further research is needed in order to extend the interaction of two emotional dimensions (valence and arousal) with the subject's gender and their effect on other bands and characteristic oscillations.

## Figures and Tables

**Table 1:** Summary of studies conducted with delta wave activity in healthy adults and patients with neurological disorders, which includes the montage method and the number of electrodes as well.

Authors	Journal	Aim of the study	Montage	Number of electrodes
Yener et al. [26]	European Journal of Neurology (2008)	Detection of subtle abnormalities of cognitive processes	Linked earlobes	13
Başar et al. [27]	Brain Research (2008)	Brain oscillations evoked by the face of a loved person	Linked earlobes	14
Harmony et al. [28]	International Journal of Physiology (1996)	EEG delta activity during difficult mental tasks and analysis of short term memory	Linked earlobes	19
Aftanas et al. [29]	Neuroscience and Behavioral Physiology (2006)	Analysis of evoked EEG synchronization and desynchronization in all frequencies bands in response to sequential presentation of IAPS pictures	Nose	62
Schürmann et al. [30]	Neuroscience Letters (1995)	A new metric for analyzing single trials ERPs based on visual P300 delta responses	Linked earlobes	7
Moretti et al. [31]	Clinical Neurophysiology (2004)	Individual analysis of EEG frequency and band power in mild Alzheimer disease	Linked earlobes	19

**Table 2:** Mean and standard deviation as a function of valence and arousal for the pictures used in the study.

	Males				Females			
Picture group	Valence		Arousal		Valence		Arousal	
	mean	SD	mean	SD	mean	SD	mean	SD

HVHA	7.41	1.51	6.59	1.98	7.1	1.7	6.0.	2.26
HVLA	6.65	1.54	3.91	2.15	6.94	1.55	3.85	2.26
LVHA	3.12	1.58	5.93	2.15	2.0	1.37	6.64	2.15
LVLA	3.9	1.53	3.91	2.04	3.6	1.54	4.16	2.1

**Table 3:** Summary of *F* values for significant effects with the ERP methodology (reported in [17]) and the delta wave methodology. Notice that both methodologies were performed on the same data, but the EROs methodology has a better sensitivity/specificity.

EROs			ERPs		
Fz	Cz	Pz	Fz	Cz	Pz

Valence by gender (*F* = 5.398)	Valence (*F* = 4.378)	Arousal (*F* = 68.51)	Valence (*F* = 9.18)	Valence (*F* = 4.23)	Arousal (*F* = 13.4)
Valence by arousal by gender (*F* = 5.439)	Arousal by gender (*F* = 5.824)		Arousal by gender (*F* = 4.96)		
Gender (*F* = 7.136)			Gender (*F* = 6.64)		
Arousal by gender (*F* = 13.519)					
